# Alport Syndrome: Clinical Utility of Early Genetic Diagnosis in Children

**DOI:** 10.3390/genes15081016

**Published:** 2024-08-02

**Authors:** Vasileia Christodoulaki, Konstantina Kosma, Nikolaos M. Marinakis, Faidon-Nikolaos Tilemis, Nikolaos Stergiou, Afroditi Kampouraki, Charalampos Kapogiannis, Vasiliki Karava, Andromachi Mitsioni, Maria Mila, Christina Kanaka-Gantenbein, Periklis Makrythanasis, Maria Tzetis, Joanne Traeger-Synodinos

**Affiliations:** 1 Laboratory of Medical Genetics, Medical School, National and Kapodistrian University of Athens (NKUA), 11527 Athens, Greece; kokosma@med.uoa.gr (K.K.); nikomari@med.uoa.gr (N.M.M.); ftilemis@med.uoa.gr (F.-N.T.); afrodite_kampouraki@yahoo.gr (A.K.); pmakryth@med.uoa.gr (P.M.); mtzetis@med.uoa.gr (M.T.); jtraeger@med.uoa.gr (J.T.-S.); 2 First Department of Pediatrics, National and Kapodistrian University of Athens Medical School, “Aghia Sophia” Children’s Hospital, 11527 Athens, Greece; nikolaosstergiou1@gmail.com (N.S.); hariskapogiannis@gmail.com (C.K.); vasilikikarava@hotmail.fr (V.K.); ckanaka@med.uoa.gr (C.K.-G.); 3 University Research Institute for the Study and Treatment of Genetic and Malignant Disorders in Childhood, Aghia Sophia Children’s Hospital, National and Kapodistrian University of Athens, 11527 Athens, Greece; 4 Department of Nephrology, “P. and A. Kyriakou” Children’s Hospital, 11527 Athens, Greece; amitsioni@icloud.com (A.M.); mnpedne@yahoo.gr (M.M.); 5 Division of Endocrinology, Diabetes and Metabolism, First Department of Pediatrics, Medical School, Aghia Sophia Children’s Hospital, National and Kapodistrian University of Athens, 11527 Athens, Greece

**Keywords:** Alport syndrome, children, RAAS inhibitors, cascade screening, *COL4A3*, *COL4A4*, *COL4A5*

## Abstract

Alport syndrome (AS) is a hereditary glomerulopathy due to pathogenic variants in *COL4A3*, *COL4A4*, and *COL4A5.* Treatment with Renin–Angiotensin–Aldosterone System (RAAS) inhibitors can delay progression to end stage renal disease (ESRD). From 2018 until today, we performed Whole Exome Sequencing (WES) in 19 patients with AS phenotype with or without positive family history. Fourteen of these patients were children. Genetic testing was extended to family members at risk. All patients received a genetic diagnosis of AS: five X-linked AS (XLAS) males, five X-linked AS (XLAS) females, six autosomal dominant AS (ADAS), and one autosomal recessive AS (ARAS). After cascade screening four XLAS males and eight XLAS females, six ADAS and three ARAS heterozygotes were added to our initial results. Fifteen patients were eligible to start treatment with RAAS inhibitors after their diagnosis. All XLAS female patients, ARAS heterozygotes, and ADAS have been advised to be followed up, so that therapeutic intervention can begin in the presence of microalbuminuria. Genetic diagnosis of AS ensures early therapeutic intervention and appropriate follow up to delay progression to chronic kidney disease, especially in thet pediatric population.

## 1. Introduction

Alport syndrome (AS) is a hereditary progressive glomerulopathy that often results in end-stage renal disease (ESRD) [1]. Apart from the renal manifestations, the patient might suffer from sensorineural hearing loss and ocular abnormalities (i.e., anterior lenticonus, posterior subcapsular cataract, posterior polymorphous dystrophy, and retinal flecks) [2]. The incidence varies from 1 in 5000 to 53,000 and is mainly due to differences in the populations studied [3]. AS occurs in 0.5% of newly developed ESRD in adults and 12.9% in children [4].

There are three types of Alport syndrome according to the mode of inheritance [4] (Table 1):

- Alport Syndrome 1: X-linked AS (XLAS), with pathogenic genetic variants in *COL4A5.*
- Alport Syndrome 2: Autosomal recessive AS (ARAS), with pathogenic variants in *COL4A4.*
- Alport Syndrome 3a: Autosomal dominant AS (ADAS), with pathogenic variants in *COL4A3.*
- Alport Syndrome 3b: Autosomal recessive AS (previously known as Thin Basement Membrane disease), with pathogenic variants in *COL4A3.*

Alport 1 occurs in 80% of all cases while Alport 2 and 3 share the remaining 20% [4].

Pathogenic genetic variants in genes *COL4A3–5* cause a defective synthesis of the a3, a4, and a5 chains of collagen type IV. These three a- chains are found in the Glomerular Basement Membrane (GBM), in the Cochlear Basement Membrane, and the base of the ocular lens [5]. This results in the heterogenicity of renal and extrarenal manifestations. In terms of renal disease, these pathogenic genetic variants produce a number of phenotypes varying from microscopic hematuria, proteinuria, to chronic kidney disease (CKD) [6].

Children often present as asymptomatic with the phenotype of early-stage AS, therefore the initiation of diagnostic investigation is triggered after an abnormal urine analysis during a routine check- up. The most common findings are isolated persistent microscopic hematuria and/or proteinuria with a mean age of presentation of 5 years. In retrospect, a positive family history (FH) will arouse suspicion of AS [7].

Clinical presentation may vary according to the type of AS (Table 1) [4,8,9,10].

Before the establishment of genetic testing, pathology findings on renal biopsy samples were essential for the diagnosis. However, this method has serious limitations, especially in children. Such limitations include the non-typical findings in GBM in the early stages of AS, the need for electron microscopy (which is not always available), and the risks that accompany the performance of an invasive procedure in children. Essentially, the non-specific findings of Light Microscopy, such as mesangial proliferation, Focal Segmental Glomerulosclerosis (FSGS), renal tubular atrophy foam cell formation, and interstitial fibrosis, are not definitely diagnostic for AS at an early stage. Electron microscopy (EM) that can reveal abnormalities of the GBM and lamination and splitting in lamina densa defines the diagnosis of AS. However, in the early stages of the disease, the only apparent finding might be the thinning of the GBM, especially in female XLAS and ADAS. Thus, it is becoming more imperative to consider genetic testing as a first-line diagnosis for AS [11].

Although there is no radical treatment for AS in children, (RAAS) inhibitors have been used efficiently for significantly delaying the progression to ESRD. The mechanism of how these medications act has been described as reducing proteinuria by decreasing filtration pressure [12]. This treatment has been proven to alter the natural course of the disease [13]. The “Updated Clinical practice recommendations for the diagnosis and management of Alport Syndrome in children and young adults 2020” established the onset of treatment at the time of diagnosis for male XLAS and ARAS patients and when microalbuminuria is present for female XLAS and ADAS patients with microalbuminuria [14]. This has contributed to highlighting the significance of early genetic diagnosis.

## 2. Materials and Methods

From 2018 until today, we have clinically and genetically diagnosed 52 cases for AS at the Laboratory of Medical Genetics, National and Kapodistrian University of Athens, Greece. The children with clinical findings suggestive of AS were referred mainly by the Pediatric Nephrology Unit of the First Department of Pediatrics, “Agia Sofia” Children’s Hospital, Athens, Greece. Three cases were referred by the Pediatric Nephrology Department of “P. and A. Kyriakou” Children’s Hospital.

The main reasons for referral were macroscopic hematuria, isolated microscopic hematuria or proteinuria, and a combination of microscopic hematuria and proteinuria. Some patients exhibited a combination of the above and/or extrarenal manifestations. In most cases (11/19), there was a positive family history (FH) of similar findings in urine analysis, extrarenal manifestations, end-stage renal disease, and a combination of the above. We have also included one case in which a pathogenic genetic variant in *COL4A3* was detected as an incidental finding. It is noted that none of our patients had received a definite diagnosis for their condition and many of them were not under any treatment.

Medical and family histories were taken, followed by clinical examination and a review of medical notes and laboratory investigations. Inclusion criteria were renal and extrarenal manifestations of AS combined with positive FH. When strong clinical suspicion was present, patients were included in the study regardless of their FH.

The method used was Whole Exome Sequencing (WES) because this secured a possible diagnosis even in cases where the clinical findings would resemble AS but the genetic variant could include pathogenic variants in genes other than *COL4A3–5*, such as *MYH9*, *LMX1B*, *LAMB2*, and *PAX2* [15]. Patients and families were investigated with WES after obtaining informed written consent and a blood sample.

WES was performed using genomic DNA extracted from whole blood. Library preparation was implemented using the Human Core Exome kit by Twist Bioscience or the Exome Solution kit by Sophia Genetics SA or IDT xGen Exome Research v2 by Integrated DNA Technologies following the manufacturer’s recommendations. The resulting libraries were subjected to paired-end sequencing on an Illumina NextSeq 500 platform. Variant annotation, filtration, and interpretation processes were performed using VarAFT v2.17 (http://varaft.eu, accessed on 2 July 2018) [16], Franklin (https://franklin.genoox.com/clinical-db/home, accessed on 1 June 2019), and Varsome Clinical platforms (https://varsome.com/, accessed on 1 June 2019) [17]. Variant filtration and classification were based on the phenotype-driven strategy [15] and according to the American College of Medical Genetics and Genomics (ACMG) guidelines [18].

A genetic WES report was communicated to the referring physician. Three possible outcomes emerged following data analysis of the results: (a) A pathogenic variant was detected in *COL4A3–5*, that could fully explain the clinical phenotype. In such cases, the families were advised to receive appropriate follow up and treatment in accordance with clinical guidelines by the nephrology department. Cascade screening was offered and performed in other family members especially where renal and extrarenal manifestations of AS were present. (b) Variants of unknown significance (VUSs) were detected in *COL4A3–5.* In these cases, parents were tested by Sanger Sequencing and the results were interpreted according to the presence and severity of their symptoms. (c) No pathogenic variant was detected. These cases are not included in the study. The patients that did not receive a conclusive diagnosis are being followed up by the Laboratory of Medical Genetics on a yearly basis and re-analysis of their WES genetic data is performed where and when appropriate.

## 3. Results

From 2018, we have collected 52 patients (n = 52), 32 adults and 20 children, most of which had a clinical phenotype of AS and were found to carry a genetic variant in one of the genes *COL4A3*, *COL4A4*, and *COL4A5.* These patients were members of 19 families (Table 2). The patients were referred from their treating physicians for genetic testing mainly because of AS renal manifestations (hematuria, proteinuria, and chronic renal disease) with or without positive family history. In cases where no other family members seemed to have symptoms of renal disease, we still chose to proceed with genetic testing. More specifically, eight cases had strong clinical suspicion for AS and WES was performed, although family history was negative. Amongst these were five cases of persistent hematuria over many years, one case where a genetic variant in *COL4A3* was an incidental finding, and two cases with significant proteinuria and a renal biopsy was performed indicating Thin Basement Membrane disease and Focal Segmental Glomerulosclerosis, respectively. The same was applied for the extrarenal manifestations of AS, such as neurosensory hearing loss and refractory anomalies of the eye. Table 3 includes the results of positive genetic variants in *COL4A3*, *COL4A4,* and *COL4A5* in our probands.

Out of 19 patients (11 females and 8 males) that were investigated, 14 were children (probands 1, 3, 6, 7, 9, 10, 11, 12, 13, 14, 15, 16, 17, and 19) aged 0.5–16 years (mean age 7 years). In two cases, although the proband was a child, we initially performed WES in the affected parental sample with a definite clinical phenotype (probands 11 and 17).

The main reason for referral was microscopic hematuria (6/19 patients), followed by hematuria and proteinuria combined (10/19 patients). Isolated proteinuria was seen in only 2/19 patients (probands 18 and 19)—one of those was of nephrotic range. All patients had hematuria and/or proteinuria with the exception of only one case (proband 13) that was investigated for dysplastic kidneys and chronic kidney disease, where a variant in *COL4A3* was an incidental finding. In the majority of patients where hematuria was present, urinalysis was positive for Red Blood Cells (RBCs) from a very young age (mean age of 5 years), with the younger patient testing positive for RBCs at the age of 5 months who shortly after developed microalbuminuria (proband 1). Positive FH was present in 11/19 cases, chronic kidney disease in 4/19 cases, and extrarenal manifestations in 5/19 cases. More specifically, the extrarenal manifestations were retinal dystrophy (proband 12), significant myopia (−8.5) (proband 17), moderate myopia (−3.5/−3.25) and astigmatism (proband 10), difficulty in swallowing (proband 16), and neurosensory hearing loss (proband 11) (Table 2).

Genetic variants in the three genes that are closely related to Alport were found in all 19 patients. Eleven patients carried a pathogenic variant in *COL4A5*, three in *COL4A4,* and five in *COL4A3* (Table 3).

After the initial genetic testing, there were five new diagnoses of XLAS males (probands 1, 2, 5, 15, and 16), six XLAS females (probands 4, 6, 8, 12, 17, and 18), seven ADAS (probands 3, 9, 10, 11, 13, 14, and 19), and one ARAS (proband 7). Following parental cascade testing, probands 18 and 19 proved to carry likely pathogenic (LP) variants, probably with reduced penetrance (or hypomorphic allele) as their parent carriers of the same variants were completely asymptomatic [19].

Cascade screening was conducted in nine families, and 28 individuals at risk were tested (Table 4). The outcome was positive for 19 of them. The remaining nine did not carry a pathogenic genetic variant of *COL4A3–5*, although among these there was one case with intermittent microscopic hematuria.

With cascade screening, we were able to identify an additional eight XLAS females, four XLAS males, three ARAS heterozygotes, and four ADAS (Table 4).

## 4. Discussion

Genetic testing is beneficial for providing definitive diagnosis, while contributing to proper usage of useful health resources [20]. In the case of AS, prompt diagnosis offers an additional benefit because of available treatment that is not curative but can alter prognosis as it dramatically delays the progression to ESRD, especially if initiated at an early stage, before there is any reduction in the Glomerular Filtration Rate (GFR) [11,21]. Children are an ideal target group for early diagnosis of AS with genetic testing, as they often are asymptomatic and the decline in GFR has not yet occurred in most cases. Thus, they benefit the most from prompt initiation of medication.

This study had dual goals, firstly in identifying new patients with AS with WES, as well as applying cascade screening to members of their family. WES can help identify possible genetic variants in *MYH9*, *LMX1B*, *LAMB2*, and *PAX2* where there is a similar clinical phenotype (proteinuria is the most profound clinical finding) and pathological findings (lamellation of the GBM) [4]. In all cases, with the exception of three (probands 7, 13, and 19), the phenotype was clearly indicative of AS. Before the establishment of genetic testing, it was thought that screening relatives at risk with urine analysis, when AS was suspected in the family, would be sufficient for early detection of the disease [11]. However, in XLAS female patients, hematuria might be intermittent [11,22]. The same applies for patients with heterozygous variants in *COL4A3* or *COL4A4* [11,23,24]. Family member cascade screening is essential because it can reveal family members at risk.

In terms of therapeutic intervention, we managed to identify several cases, amongst which were a few children that were eligible to instantly start treatment or be closely monitored for the presence of microalbuminuria [14]. Amongst our cohort, we identified an additional six patients that were eligible to immediately start treatment with RAAS inhibitors. From this group of 10 patients (9 XLAS males and 1 ARAS), only half were already on this treatment. Three female XLAS and two ADAS with microalbuminuria were also strongly advised to receive treatment. So far, all our pediatric patients have normal renal function with serum creatinine and eGFR within a normal range. Those that already had microalbuminuria have responded well to RAAS inhibitors with the exception of proband 7 that receives both an ACE inhibitor and Angiotensin II AT 1 receptor antagonist and still has proteinuria of 1 gr/24 h.

Studies from previous years show that XLAS female patients [25] and ARAS heterozygotes [26] were thought to be only carriers of the disease and quite often were discharged from follow up. What we now know is that they can also advance to ESRD [19,27]. Proband 17 is such an example and was diagnosed with AS after WES testing. We note that she presented to our clinic with her 13-year-old son that was monitored for intermittent microscopic hematuria. When taking FH, proband 17 proved to have hematuria, proteinuria, chronic renal disease, and high myopia. Genetic testing was performed on her, because of AS phenotype suspicion, and the genetic variant c.1871G>A, p.(Gly624Asp) in *COL4A5* was identified. Up to that point, she was treated as a chronic kidney disease patient of unidentified cause, and she had also undergone a renal biopsy with pathological findings of interstitial nephritis and Thin Basal Membrane disease. Her son carried the same variant and was advised to begin treatment with RAAS inhibitors at the time of genetic diagnosis. This example illustrates the significance of cascade screening and how inheritance pattern (in this case XLAS) can guide follow up and management when appropriate. In our cohort, we identified 14 XLAS female patients that now receive proper treatment and follow up. This indicates that the most significant achievement of this study was the identification of the cases that would not have received treatment promptly had it not been for genetic diagnosis. Moreover, some of the XLAS male patients presented only with microscopic hematuria and had no clinical indication for treatment. This was only started after AS diagnosis and is bound to alter the natural course of the disease and offer them better prognosis, which is even more important in the cases of probands diagnosed at a younger age and having normal GFR.

Another example is proband 3 (ADAS) who presented with microscopic hematuria. This patient’s mother was found to have the same genetic variant, suffer from ESRD, and is on dialysis. The maternal grandmother who also has raised creatinine, hematuria, and proteinuria was also tested and has the same variant. After these results, our patient (proband 3) was advised to be monitored very closely and start treatment as soon as she develops microalbuminuria.

Also, ARAS heterozygotes that would until recently not have a diagnosis of renal disease are currently under follow up and will be given RAAS inhibitors in the event of microalbuminuria. An ARAS carrier of c.1598G>A, p.(Gly533Asp) in *COL4A4*, had been initially diagnosed with IgA nephropathy due to non-specific pathological findings in a renal biopsy performed due to hematuria and proteinuria [28].

Cascade screening was not difficult to implement because in most cases, relatives were willing to be tested, especially when they experienced symptoms themselves and were hoping for a diagnosis/treatment, or when they were at reproductive age and planning to start or expand their family. We did, however, encounter logistical difficulties such as the cost of the test since it is not covered by state insurance and family relations.

Ten patients with positive genetic testing had undergone renal biopsy a few years earlier. Six of these consisted in our main probands that were initially tested with WES (probands 2, 11, 16, 17, 18, and 19) and the remaining four were part of cascade screening. Renal biopsy results in the first group were as follows: Secondary Focal Segmental Glomerulosclerosis (FSGS), and Thin Basal Membrane disease (six cases). In the second group, pathological reports were as follows: AS, Thin Basal Membrane disease, and non-specific findings of Mesangial Proliferative Glomerulonephritis (four cases). However, in most cases, the renal tissue had not been examined by electron microscopy (EM), mainly because this was not available at the time. Renal biopsy is a highly invasive procedure that is often followed by complications and requires a hospital stay. We therefore remain reluctant in applying it as a diagnostic tool especially in children. Adding the non- specific diagnosis that we often obtain in AS, it is becoming increasingly clear that genetic testing is the method of choice for AS [28]. Our patients received the genetic diagnosis of AS that hopefully will lead to more targeted treatment avoiding unnecessary immunosuppressant medications that are often given in this group of patients. Cyclosporin has proven to be non-beneficial in AS with the phenotype of FSGS [29].

In the case of proband 12 with c.1871G>A, p. (Gly624Asp), there was the extrarenal manifestation of retinal dystrophy which, interestingly enough, was due to an additional genetic variant in *TGFBI* (c. 370C>T, p.(Arg124Cys)). This case underlines that WES is the method of choice to diagnose AS, as GenePanel testing has the risk of missing additional variants responsible for complex phenotypes, as was the case for the specific proband whose retinal dystrophy might have been attributed to AS and therefore would receive the wrong follow up, treatment, prognosis, and genetic counseling.

Even though we only had 19 probands, we nonetheless found some common genetic variants: c.1871G>A, p.(Gly624Asp) in four cases; c.1006G>T, p. (Gly336Cys) in two cases; and c.1321_1369+3del in another two cases.

Last, in the individuals with no genetic findings, it was felt that there was a clinical benefit as the result altered their follow up by reducing unnecessary laboratory testing (i.e., urinalysis) and, in the case of one individual with persistent microscopic hematuria, to guide follow-up and investigation towards non-genetic diagnoses [27].

Moreover, promising new therapeutic agents that have evolved from current clinical trials include Endothelin Type A Receptor (ETAR) and Angiotensin II Type 1 Receptor (AT1R) inhibitors, lipid-modifying drugs, Discoidin Domain Receptor 1 (DRR1) inhibitors, Osteopontin Blocking Agents, and genome editing therapies, and they remain to be assessed further for their safety and efficacy in the child population [30]. Thus, genetic testing in children will eventually, if it has not already, become the gold standard for the diagnostic approach of AS.

## 5. Conclusions

This study underlines the importance of using WES as a first line of genetic testing in patients with the spectrum of phenotypes compatible with Alport syndrome because an accurate diagnosis is very important for the appropriate follow up, treatment, and genetic counseling, especially in children. Moreover, early genetic diagnosis improves quality of life for the patients and their families, mainly by removing uncertainty.

As there are limited data from long-term treatment with RAAS inhibitors for AS in children, more multicenter perspective studies should be performed. We aim to continue the follow-up of our patient cohort, anticipating more positive outcomes, by identifying new cases and by expanding cascade screening to all family members at risk.

## Figures and Tables

**Table 1:** Clinical presentation of AS according to genotype.

Mode of Inheritance	Genes Involved	Renal Manifestations	Extrarenal Manifestations	Age of ESDR	Positive Family History (FH)
XLAS Alport Syndrome 1	*COL4A5*	Microscopic Hematuria -**100% of cases of male patients** -98% of female patients Microscopic hematuria and proteinuria—73% of all patients **In male patients, proteinuria is often profound from an early age**	Sensorineural hearing loss by the age of 40 -90% of males -12% of females Ocular abnormalities Leiomyomas	90% by the age of 40 in male patients 12% by the age of 40 in females	Hematuria and/or proteinuria (15% de novo variants)
ARAS Alport Syndrome 2	*COL4A3* and *COL4A4*	Similar to XLAS in both genders	Sensorineural hearing loss at mean age of 20 years	21 years of age	Carriers often asymptomatic -microscopic hematuria and mild proteinuria
ADAS	*COL4A3*	Proteinuria presents later in life (mean age 17 years)	Sensorineural hearing loss and ocular abnormalities rarely occur	70 years of age	Usually, positive

**Table 2:** Distribution of probands in relation to most common referral reasons and number of cases with positive family history, chronic kidney disease, and extrarenal manifestations.

Reasons for Referral	Isolated Proteinuria	Isolated Hematuria	Combination of Proteinuria and Hematuria	Extrarenal Manifestations	Chronic Kidney Disease	Positive Family History
Number of probands-patients	2/19	6/19	10/19	5/19	4/19	11/19

**Table 3:** Genetic variants and phenotype.

Proband	Gender	Age at Diagnosis	Gene	Genetic Variant	ACMG/ACMG and ClinGen Classification (for CNVs)	Phenotype
1.	Male	10 months	*COL4A5* (Exon 15)	c.845dupG p.(Gly283Trpfs*2)	Pathogenic PVS1, PM2, PP3	XLAS
2.	Male	36	*COL4A5* (Exon 24)	c.1589G>A p.(Gly530Asp)	Pathogenic PM1, PM2, PP2, PP3, PP5	XLAS
3.	Female	12	*COL4A3* (Exon 10)	C575G>A p.(Gly192Glu)	Likely pathogenic (PM1, PM2, PP3, PP4)	ADAS
4.	Female	24	*COL4A5* (Exon 25)	c.1871G>A p.(Gly624Asp)	Pathogenic (PS4, PM1, PM5, PP3, PP5)	XLAS
5.	Male	52	*COL4A5* (Exon 28)	c.2228G>T p.(Gly743Val)	Likely pathogenic (PM1, PM2, PM5, PP2, PP3)	XLAS
6.	Female	5	*COL4A5* (Exon 3)	c.219_226del p. (Arg74Lysfs*3)	Likely pathogenic (PVS1, PM2)	XLAS
7.	Female	12	*COL4A4* (Exon 20 Exon 22)	c.1598G>A p. (Gly533Asp) c.1321_1369+3del	Pathogenic	ARAS
8.	Female	17	*COL4A5* (Exon 47)	c.4359delT p.Gly1454Glufs*94	Pathogenic PVS1, PM1, PM2, PP2	XLAS
9.	Male	15	*COL4A4* (Exon 20)	c.1321_1369+3del	Pathogenic PVS1, PM2, PP4, PP5	ADAS
10.	Female	9	*COL4A3* (Exon 18)	c.1006G>T p. (Gly336Cys)	Pathogenic (PS4, PM1, PM2, PP3, PP5)	ADAS
11.	Male	53	*COL4A3* (Exon 19)	c.1087G>T p.(Gly363Trp)	Pathogenic (PM1, PM2, PM5, PP1, PP4, PP3)	ADAS
12.	Female	3	*COL4A5* (Exon 25)	c.1871G>A p. (Gly624Asp)	Pathogenic (PS4, PM1, PM5, PP3, PP5)	XLAS
13.	Female	5 months	*COL4A3* (Exon 3)	c.172 G>A p. (Gly58Ser)	Likely pathogenic (PM1, PM2, PP3, PP4)	ADAS or ARAS
14.	Male	10	*COL4A3* (Exon 18)	c.1006G>T p. (Gly336Cys)	Pathogenic (PS4, PM1, PM2, PP3, PP5)	ADAS
15.	Male	5	*COL4A5* (Exon 25)	c.1871G>A p. (Gly624Asp)	Pathogenic (PS4, PM1, PM5, PP3, PP5)	XLAS
16.	Male	16	*COL4A5* (Exon 39)	c. 3508G>A p.(Gly1170Ser)	Pathogenic (PS4, PM1, PM2, PM5, PP2, PP3, PP5)	XLAS
17.	Female	49	*COL4A5* (Exon 25)	c.1871G>A p.(Gly624Asp)	Pathogenic PM1, PP2, PP3, PP4, PP5	XLAS
18.	Female	20	*COL4A5* (Exon 24)	c.1603C>T p.(Pro535Ser)	Likely pathogenic (PM1, PM2, PP2, PP3)	XLAS
19.	Male	2	*COL4A4* (Exon 36)	c.3349C>A p.(Prol117Thr)	VUS PM2	ADAS

**Table 4:** Types of AS after completion of cascade screening in 19 families (52 individuals).

Types of AS after Genetic Testing	Number of Probands (WES)	Number of Individuals That Had Positive Genetic Findings from Cascade Screening	Total Number
XLAS males	5	4	9
XLAS females	6	10	16
ADAS	7	7	14
ARAS	1	3 heterozygotes	4
Total number	19	24	43

## Data Availability

The datasets used and/or analyzed during the current study are available from the corresponding author upon reasonable request.
